# The interplay of social identity and norm psychology in the evolution of human groups

**DOI:** 10.1098/rstb.2021.0412

**Published:** 2023-03-13

**Authors:** Kati Kish Bar-On, Ehud Lamm

**Affiliations:** ^1^ The Science, Technology, and Society program, Massachusetts Institute of Technology, Cambridge, MA, USA; ^2^ The Cohn Institute for the History and Philosophy of Science and Ideas, Tel Aviv University, Tel Aviv 69978, Israel

**Keywords:** social identity, norm psychology, human evolution, group behaviour

## Abstract

People's attitudes towards social norms play a crucial role in understanding group behaviour. Norm psychology accounts focus on processes of norm internalization that influence people's norm-following attitudes but pay considerably less attention to social identity and group identification processes. Social identity theory in contrast studies group identity but works with a relatively thin and instrumental notion of social norms. We argue that to best understand both sets of phenomena, it is important to integrate the insights of both approaches. Social status, social identity and social norms are considered separate phenomena in evolutionary accounts. We discuss assumptions and views that support this separation, and suggest an integrated view of our own. We argue that we should be open to the early origins of human social complexity, and conjecture that the longer that the human social world involved multi-level societies the more probable it is that norm psychology and social identity interacted in rich ways.

This article is part of the theme issue ‘Human socio-cultural evolution in light of evolutionary transitions’.

## 1. Introduction

The structure of human groups has changed, developed and evolved throughout history. Unlike beehives or termite mounds, recent transitions in the evolution of human groups are not primarily biological; instead, culture and the establishment of social institutions are responsible for setting such transitions in motion [1,2]. The emergence of social institutions and social norms has played a particularly significant role in shaping group behaviour and individuals' norm-following attitudes [3]. The current paper focuses on two prominent theories that deal with group behaviour: norm psychology and social identity theory (SIT), and aims to evaluate the possibility that to adequately understand the psychological underpinnings of peoples' adherence to social norms we must simultaneously take into account both theories. It is thus an attempt to integrate two hitherto separate scientific approaches to the unique features of human sociality.

The label norm psychology was introduced to refer to the suite of psychological adaptations involved in humans' capacity to establish and enforce social norms and behave according to the social norms of their society [4]. Accounts of norm psychology have paid considerable attention to the notion of norm internalization and the role of norm internalization processes in shaping human behaviour [4–10]. However, such accounts hardly ever address individuals’ social identity and the way it affects group identification, as well as its impact on norm-following behaviour and attitudes. Group belonging and identification are, for the most part, taken as a given.

SIT was introduced earlier to explain people's identification with social groups [11–14]. Subsequent work addressed how groups differ in their attitudes towards norms and norm-following, and how people systematically differ in their attitudes toward different sets of social norms and are more conformist or attached to some sets of norms than to others. That said, as we will show, SIT applies a relatively narrow definition of social norms, usually conceived as collective beliefs about appropriate behaviour. Considered separately, work on norm psychology and SIT each portrays a partial picture of a single phenomenon; when considered together, they have the promise to provide a richer understanding of individuals' attitudes towards norms and the evolution of norm compliance. However, such an integration is not straightforward and may go against some of the assumptions underlying these separate scientific endeavours. Evaluating its prospects and suggesting a way forward is the aim of this paper.

Accounts of human evolution distinguish between social hierarchy and in-group/out-group behaviour in terms of timing and theory. Social hierarchy may be connected to the type of social hierarchy found in apes (i.e. *Pan*), possibly including prestige hierarchy in humans [15,16]. A related issue to the psychology of hierarchy and status from an evolutionary perspective is the idea of social roles [17,18]. Possibly later, humans evolved in-group/out-group psychology and norm psychology, perhaps as a result of cultural group selection [19–21]. Another possibility is that the early evolution of culturally integrated societies derived from processes of coevolution of institutions (such as language), biological systems and cultural evolutionary individuals [22,23]. The integration of norm psychology and SIT that we conjecture raises questions and difficulties with this standard division. This has potential implications for understanding the phenomenology of social behaviour, determining the timing and evolutionary sequence, and evaluating evolutionary explanations of these phenomena.

Our discussion is organized as follows. In §2, we briefly present SIT and discuss the role social norms play in it. Section 3 surveys several accounts of norm psychology and points out tensions between norm psychology and SIT. Section 4 analyses the differences between the two approaches and §5 sketches the outlines of an account that combines insights from both norm psychology and SIT. Section 6 concludes with preliminary remarks about the implications of our arguments for understanding in-group and out-group interactions in complex social environments, such as contemporary multi-cultural societies, and possible implications for thinking about the evolution of human sociality.

## 2. The role of social norms in social identity theory

In the second half of the twentieth century, several competing theories of social norms emerged. The sociologist Talcott Parsons argued that people adhere to norms because they are internalized in childhood and henceforth operate as personal needs that people strive to satisfy [24]. SIT was developed in the early 1970s, primarily by the social psychologist Henri Tajfel and his colleagues. Their theoretical ideas were grounded in the minimal-group experimental paradigm. In these experiments, people seemed to immediately identify with arbitrary groups and establish in-group and out-group attitudes even towards random, meaningless and temporary group assignments. Tajfel's student John Turner later connected this work to various cognitive factors that affect how people perceive group identity and social position and determine behaviour (e.g. stereotyping). In her book, *What are Norms?,* the sociologist and anthropologist Francesca Cancian differentiated between Parsons' approach (namely, the socialized actor theory) and SIT [12]. According to the latter, norms are not internalized but are rather shared conceptions about the roles and ranks in the community. Cancian defined social norms as shared beliefs about which actions and attributes bring respect and approval (or disrespect and disapproval) [12, p. 6]. She marshalled anthropological evidence that supports the claim that individuals conform to social norms to validate their social identity without internalizing them. She did so in the book's last chapter by showing that people change their norms very quickly when they become members of other groups with different beliefs and norms. We will later argue that people's ability to quickly move between groups and social contexts is critical for understanding norm psychology. While in Parsons' view norms are internalized as part of socialization and hence norm change is very slow, according to SIT people change their norms when their social identity changes, and both can happen quickly.

Tajfel defined social identity as ‘that part of an individual's self-concept which derives from his knowledge of his membership of a social group (or groups) together with the value and emotional significance attached to that membership’ [13, p. 255]. He argued that when people voluntarily categorize themselves as belonging to a specific group, they perceive themselves differently, and their self-conception changes.

Tajfel's doctoral student and the developer of Social Categorization Theory, the psychologist John Turner, made the distinction between *social identity*, which refers to self-definition in terms of group memberships, and *personal identity*, which refers to self-descriptions in terms of personal and idiosyncratic attributes [14].^1^ According to Turner's interpersonal–inter-group continuum, social identity and personal identity are two distinct types of self-categorization [28]. However, as Bicchieri, Muldoon and Sontuoso point out, the two levels often interact and influence each other, and hence the distinction between them must be taken only as an approximation [11].

Tajfel and Turner distinguished between three mental processes that occur when people classify others as belonging to their in-group or out-group: social categorization, social identity and social comparison [29]. By social categorization, they refer to the way people use social categories and assign themselves and others to a category they believe they belong to. After being socially categorized, people adopt the identity of the group they categorize themselves as belonging to and conform to the norms of the group. This mental process is dubbed social identity by Tajfel and Turner. The third mental process, which they called social comparison, refers to how people compare their own group with other groups in order to maintain a feeling of superiority over an out-group.

It is important to note that in SIT, group membership is not something exogenous, which is attached to the person, but rather an endogenous and vital part of the self. By contrast, while social norms play a significant role in SIT, they are often described solely as attributes of the group or as signals of social identification. Put differently, according to SIT, the motivation a person has to adhere to a certain social norm derives from a desire to validate his identity as a group member, and it does not involve internalization of that specific norm in the sense of personal psychological commitment to the content of the norm. Recent discussions about the role of social norms in explaining human behaviour portray quite a different picture by shifting the focus to individuals' norm psychology. We now turn to a brief examination of these approaches.

## 3. Social norms and norm psychology

The notion of norm psychology was proposed to describe the psychological underpinnings of norm-governed behaviour [4]. These authors defined norm psychology as a characteristic of individuals' psychology that describes their ability to acquire, implement and participate in a norm-governed society. Other researchers share this general perspective [7–9,30,31].

A large number of theoretical and experimental studies employ diverse approaches to humans' unique phenomenon of norm abidance, commitment and enforcement; we will mention only a few. Each embraces a different definition of what social norms are or operationalizes them differently, but all of them build upon the somewhat vague concept of social norms. While they have different explanatory goals and methodological commitments, they all address social norms as having a central theoretical role yet do not pay much attention to the connection between social identity and norm psychology. Nonetheless, each of them at least mentions the concepts of social identity or group identity in their work.

The developmental psychologist Michael Tomasello defines social norms as ‘socially agreed-upon and mutually known expectations bearing social force, monitored and enforced by third parties’ [31, p. 87] and suggests that we are genetically endowed with a predisposition for sociality that is shaped and developed through a process of socialization [9,31]. According to Tomasello, children's ability to learn, follow and enforce social norms ‘reflects not only humans' special sensitivity to social pressure of various kinds, but also a kind of group identity and social rationality’ [31, p. 44]. If a person wants to be a member of a certain group, they must follow the group's norms [9, p. 119]. Tomasello refers to the roots of this process of commitment as ‘generalized normativity’ that ‘ends up back at group identity’ [9, p. 119], in the sense that group identity is the driving force behind people's commitments.

The cultural psychologist Michele Gelfand views social norms as ‘rules for acceptable behaviour’ that hold groups together, ‘give us our identity’ and ‘help us coordinate in unprecedented ways’ [6, p. 11]. Gelfand presents evidence that individuals' norm psychology is closely tied to their culture being tight or loose, where latitude or constraint in the cultural context affects the psychological characteristics of individuals [6,32].

The philosopher Christina Bicchieri sees norm-governed behaviour through the general framework of rational choice and defines a social norm as a ‘rule of behaviour such that individuals prefer to conform to it on condition that they believe that: (i) most people in their reference network conform to it (empirical expectation) and (ii) that most people in their reference network believe they ought to conform to it (normative expectation)’ [5, p. 35]. Bicchieri defines sensitivity to a norm as the degree to which a person adheres to what the norm stands for [5, p. 165]. It embodies one's personal reasons and inner motivations to comply with a norm and may be subject to change depending on one's sensitivity to pressure to conform or when new information emerges. Bicchieri claims that in cases of competing norms, people's norm-following attitudes are shaped by their normative expectations as well as by the norms they perceive as reinforcing their identity. She illustrates this with the case of condom use by men in which norms of masculinity and norms of responsibility push in opposite directions. A person may justify his refusal to use condoms by deciding that ‘masculinity norms, which are shared, justified and approved by his buddies, are more important to his identity’ [5, p. 104].

According to Chudek and Henrich's account of norm psychology, which builds on work on groups in cultural evolution, mechanisms for sustaining cooperation and other norms operate within groups and affect inter-group competition, and this selects for psychological adaptations for norms [4, p. 220]. One of the key components of norm psychology is the ability to acquire norms. In turn, these psychological adaptations for norms facilitate cooperation within the group and competition between groups. Recently, Sterelny expressed scepticism about this model, which is based on cultural group selection and originated in the earlier work of Boyd & Richerson [19] and Henrich [20], and argued for a more individualistic account [21, p. 84]. Sterelny claims that norms emerged late, in humans pretty much like ourselves. These humans have had a long history of associations and collaborations, and hence, we would argue, at least the beginnings of complex social identities.

In contrast with views that take the acquisition of norms to be fundamental for group identity, in SIT social identity is the basis of group behaviour and underlies norm acquisition. According to Tajfel, the group provides its members with a positive social identity, in the sense that it makes the individual value the distinctiveness of his own group compared to other groups [33]. Group identification becomes a salient part of one's identity, and in order to secure and reinforce their social identity, individuals define themselves in terms of the group that they see themselves as belonging to by adopting and adhering to the group norms. Moreover, experiments show that as group identity becomes more salient and of intrinsic value, individuals tend to behave according to the group rules and exercise personal restraint in cases of conflict between group identity and personal identity [34,35]. The juxtaposition of norm psychology and SIT side by side gives rise to the question of what exactly is being internalized and when. On the one hand, norms are internalized to the point they become goals in themselves, comprising a significant part of an individual's identity. On the other hand, social identification processes render group identity an integral part of people's identity. That is not to say that processes of norm internalization are not part of SIT; they certainly are. However, in SIT, norm internalization processes do not receive the primacy that norm psychology accounts assign to them.

## 4. Internalization

The notion of intrinsic motivation refers to the fact that norms become goals in themselves or part of individuals' utility functions and motivate action regardless of other payoffs and sanctions [7,8,36,37]. The process of internalization refers to one possible explanation of how norms are acquired and come to play such a role. Processes of norm internalization begin in early childhood, and internalized norms become a significant part of a person's identity, making it extremely difficult to change an individual's internalized norms. Many theories of norm psychology maintain that individuals' attitudes towards social norms originate from such a process that leads to lifelong commitments to specific social norms ([7–9]; for historical and theoretical context, see also [37,38]). Norm internalization helps people navigate the social landscape and reduces the costs associated with evaluating the costs and benefits of adhering to social norms. It has been argued that natural selection built us to be norm internalizers and that internalized motivations help us avoid temptations to break the rules [7,37,39–41]. However, internalization implies less behavioural flexibility [7,36].

Christina Bicchieri considers the process of norm internalization to be related to the individual's process of the developing of moral beliefs corresponding to societal standards [5]. Emotions like guilt then support the motivation to conform to these norms. However, Bicchieri also emphasizes that behaviour is the outcome of rational decision making, in which internalized norms are but one factor affecting actors' decisions.

In contrast with these accounts of norm internalization, SIT suggests that people's motivation to adopt certain norms derives from their desire to confirm their social status within their group's social hierarchy. Norms are thus not irrevocably internalized; they readily and rapidly change with changes in group memberships, social status and social context. According to Turner's social categorization theory, individuals undergo a process of norm internalization, but only after they define themselves as members of a distinct social category and learn or develop the category or group's appropriate and expected behaviours [28]. This happens through processes of depersonalization and self-stereotyping [14,28]. Thus, norm internalization is affected by the degree to which individuals consider themselves to be members of the group. This description is significantly different from that of norm psychology accounts. Unlike norm psychology, in SIT, the individual's attitude towards social norms is determined by her level of identification with the group. She follows norms not because she internalized them but because she wants to secure her social identity. In turn, her behaviour according to her group's norms contributes to the process of norm internalization. According to norm psychology, the course of events is the opposite: the individual's attitude towards social norms is determined by norm internalization. Through the process of norm internalization, the individual maintains her status as an in-group member, not the other way around.

The two pictures offered by the two approaches are as follows. According to internalization views, the content of norms is acquired through internalization and rarely changes, and individuals are psychologically committed to the norms so acquired. Social behaviour, in turn, is affected by a person's social norms as well as the specific social context that they find themselves in. According to a more dynamic picture suggested by SIT, the content of norms may be directly determined by the current social context. Moreover, social behaviour may change the content of norms and psychological commitments. While sophisticated internalization views provide explanations of how and when behaviour may go against a person's norms (for example, because of expectations about the behaviour of others), they pay little attention to how this change in behaviour may affect individuals' commitments and to the psychological tension that can arise when commitments and behaviour clash.

An interesting case, we suggest, involves the process of social identification. According to SIT, people change their norms or norms-following attitudes to protect their social status. Norm-governed behaviour may be affected in different ways depending on whether a person judges the people around her as belonging to her in-group, her out-group, or her desired in- or out-group. A change in social circumstances may affect her social status, and in order to maintain or improve her social position, she may adopt new behaviours and norms. According to SIT, the purpose of the change of norms is to validate social identity and status, and she will change them again if she feels that her social identity is threatened. The anthropologists Jean Ensminger and Joseph Henrich and their colleagues concluded from their rich studies that people have many conflicting internalized goals and motivations, and they have to determine their behaviour according to all [42]. Among those internalized goals, possibly high on the list, we suggest, are social identity and group identity.

SIT and norm psychology accounts have different interpretations of humans’ acquisition of norms and of norm-governed behaviour. However, those interpretations are not mutually exclusive but complement each other. To better understand the relations between the two theories within the broader context of human behaviour, we now sketch an integrated approach that takes both theories into account.

## 5. Towards an integrated approach of norm psychology and social identity

The idea that social identity affects norm-governed behaviour and vice versa has been tackled from different angles by several studies in psychology, political science, philosophy, economic decision making and cultural evolution studies [43–49]. It has been argued that the structure of social identity is tied to the structure of society, that social identity affects cooperation between individuals [50], that norm-following behaviour can reinforce group identity [51], and that social norms are not individual-level but community-level patterns of social behaviour [52].

However, as Davis and Kelly rightfully point out, the absence of a comprehensive conceptual overview of the different approaches and their interactions stands out [48]. We aim to take a step towards creating such a framework by suggesting an integrated account of norm psychology and social identity. Norm psychology theories focus on different psychological characteristics to SIT. Nonetheless, as we have seen throughout §§3 and 4, each approach acknowledges the relevance of the other, even if only as a marginal sidekick. Integration between the two approaches will provide a fuller picture of group behaviour as driven by evolution, society, culture and personality traits. Addressing social identification processes as significant components of people's cognitive mechanisms that influence their attitudes towards social norms adds to the accounts offered by norm psychology for people's rapid change of norms. On the other hand, norm psychology approaches present a rich account of social norms and norm internalization processes that receive considerably less attention in SIT and situate the discussion within an evolutionary framework.

Individuals may be committed to a norm that they acquire to varying degrees, which manifest as the degree of intrinsic motivation they have to act according to the norm (see [5]). SIT would be enhanced by addressing social norms as goals in themselves, to which people feel committed to some degree. Thus, people behave in accordance with their group norms since they internalize both social identity and social norms and feel obligated to both. People's ability to recognize and punish norm violators is affected by the internalization of social norms. However, it is also affected by the internalization of social identity, which favours group identity and makes group members suspicious towards people who do not obey the norm, and affects which norms are salient in a given social context. It is significant that probably the most common form of punishment for norm violation, often found in foragers, is social pressure, affecting the social status of the violators, for example, using humour and gossip to make fun and bring down status seekers. Therefore, norm psychology theories would likewise be enhanced by taking social identity factors into account.

**Figure 1.**
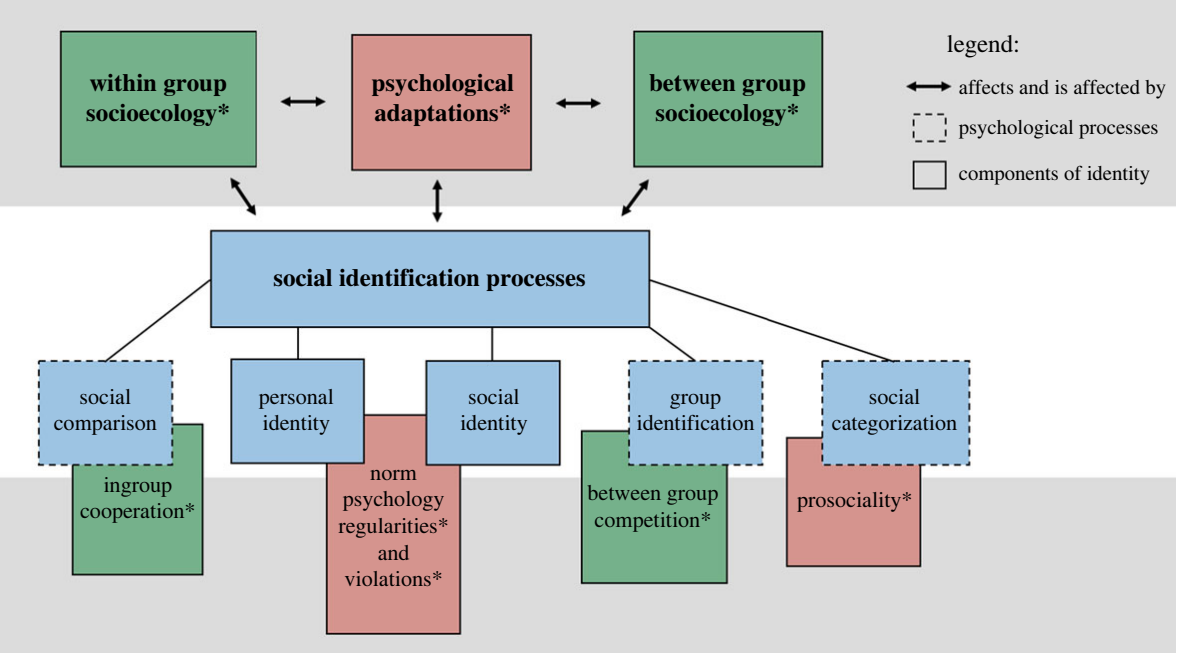
Key connections between SIT and norm psychology. Boxes represent components, and their colour represents the components' origin: blue for components that are part of SIT, green for Chudek & Henrich's group socioecology and red for psychological adaptations. Overlapping boxes suggest that their components interact with each other and that one component cannot be fully understood without the others. Components marked with an asterisk are taken from Chudek & Henrich [4]; elements with a grey background are taken from Chudek & Henrich [4]; elements with a white background represent the components and connections our integrated approach contributes to their analysis. (Online version in colour.)

**Figure 2.**
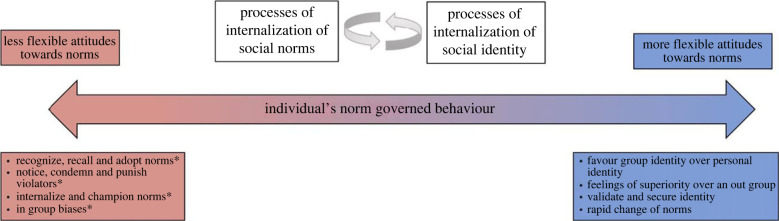
The behavioural continuum of an individual's norm-following attitudes, as affected by processes of norm internalization (red) and internalization of social identity (blue). Following norms because of personal commitment and intrinsic motivation is depicted on the left-hand side of the continuum; adjusting which norms are activated and adjusting behaviour based on social context and considerations of social identity is depicted on the right-hand side of the continuum. All entities marked with an asterisk are taken from Chudek & Henrich [4]. (Online version in colour.)

The central idea of our proposed account is described in figures 1 and 2 below. Figure 1 portrays the mutual interactions between the two theories by highlighting the components from norm psychology that shape and are shaped by SIT and vice versa. Rather than limiting our attention to the early internalization of norms, we emphasize that acquiring and activating norms are dynamic processes that occur throughout most human life. Cooperation, group competition, pro-sociality and humans' ability to detect regularities and violations of norms affect social identification processes. Similarly, group identity, social comparison, social status and personal identity affect norm internalization and activation processes. For example, if social circumstances are such that individuals constantly need to validate and secure their identity, they may tend to change their norms or adjust the demands of their norms more rapidly and have more flexible attitudes towards them. However, if the social context is relatively stable and individuals feel that their social status is secure, they would tend to follow the norms they already acquired, feel more committed to them and generally have less flexible attitudes towards norms change and norm transgressors.

The outcome of the constant interaction between the two internalization processes (of social norms and social identity) is depicted in figure 2. People's norm-governed behaviour resides on a continuum, ranging from flexible attitudes towards norms to less flexible attitudes. The ability of people to recall and adopt norms and to notice and condemn norm violators is influenced by processes of social identity, and their ability to favour group identity or change norms when it is socially necessary or helpful is influenced by processes of norm acquisition and activation. Depending on the social context which affects the salience or activation of certain sets of norms, people's norm-governed behaviour may be more or less flexible. Likewise, depending on this context, both norm content and social identity may be adjusted by the agent. This adjustment, we suggest, is the result of the mutual influence that processes of norm internalization have on social identity and that processes of social identity have on individuals' norm-governed behaviour.

The proposed account highlights where the two different perspectives of social identity and norm psychology conflict and how they reinforce one another. Specifically, it highlights that internalization of social identity and internalization of social norms are processes that interact constantly and are hard if not impossible to separate. Our integrated view of norm psychology and social identity suggests that when social circumstances change, people adapt by re-evaluating their social status and, as a result, may change their norm-following attitudes. They behave according to the norms they internalize but also adjust their behaviour to fit their social identity and group identity. Social identification processes determine group belonging, and group identification processes determine which norms became salient. It is, therefore, not only that processes of social identification and norm internalization affect each other, but that together, these two invariably interacting processes shape peoples' adherence to social norms.

This observation highlights the importance of considering carefully the type of groups and intra-group organization within which norm psychology and social identity evolved [53–57]. Multi-level societies composed of family units of close kin, extended families, foraging units and other social units give rise to the possibility of shared social norms and social identities, as well as a whole range of degrees of conflict between people with overlapping but distinct norms and identities. Modern human hunter–gatherer societies are multi-level societies, and multi-level societies may have been typical for a long span of human evolution [1,57–60]. In contemporary foraging societies, bands are nested within communities of about 150–500 adults, and such communities are part of ethnolinguistic groups numbering a few thousand. Forager bands have fluid membership compared to *Pan* groups, and what is even more telling for the issues discussed in this paper is that these communities assemble periodically, bringing into contact members of several bands [21]. In these situations, multiple social identities are simultaneously active since individuals do not forget the smaller social units that they also belong to. A further clue regarding the social negotiation individuals experience is that many individuals are multi-lingual [61]. A possible evolutionary implication may have been the importance of the ability to coordinate and negotiate between norm systems, issues that are best understood by combining the insights of norm psychology and SIT.

## 6. Concluding remarks

This paper argues that an adequate account of human normative behaviour must integrate insights from both norm psychology and SIT. Each of the two approaches sets forth to explain humans' norm-following attitudes and group-related behaviour, and each addresses a specific aspect that the other one lacks. Norm psychology accounts focus on social norms and norm internalization processes, but they pay little attention to processes of social identification and their impact on individuals' commitments to their group, wherein norms allegedly come from. Conversely, SIT concentrates on group identification and social categorization processes. However, its analysis of social norms is mostly instrumental in the sense that social norms and adherence to social norms are merely indicators or signs of an individual's degree of commitment to the group rather than the result of his commitment to the content of specific norms. Moreover, it does not provide an evolutionary perspective on the origins of human norm psychology.

The sociologist John Finley Scott pointed out that ‘the term internalization is a metaphor: it implies that something moves from outside the mind or personality to a place inside it’ [62, p. 3]. Combing insights from both approaches, we suggested that this metaphor can be unpacked by noting that it applies to two different factors: social norms and social identity, and argued that the *interplay* of both affects behaviour.

Internalization of social norms and internalization of social identity can be seen as two complementary processes, both playing a role in norm-governed behaviour. They both influence and shape the development of an individual's attitudes towards norms and norm-following, as well as their feelings of commitment to their group. Furthermore, both do not necessarily end after childhood but rather depend on complex social negotiation and movement between social groups.

One research area that may benefit from the integration urged in this paper is the evolution of human groups. Norm psychology is among the most important sets of mechanisms underlying human societies [30,37,39–41,53–55,63]. Another crucial factor is the predisposition to impose social categories that produce group boundaries and identities [53]. We showed how these processes depend both on the factors studied under the heading of norm psychology and on factors studied under the heading of SIT—and, significantly, argued that the two issues are almost entirely inseparable.

Evolutionary accounts of norm psychology should address the interaction between social norms and social identity. As we noted in the introduction, social identity and norm psychology are not only separate due to the vagaries of intellectual history. Their divorce is now grounded in arguments about their evolution. The psychology of human social hierarchy, perhaps in the form of prestige, arguably evolved earlier, in the context of small, non-interacting groups. By contrast, the psychology of norms mostly evolved later, possibly due to interactions between groups (see [21] for a differing view). This, however, does not imply that the psychological mechanisms that evolved are separate and independent. Psychological research on contemporary humans, of the sort reviewed above, suggests that they are not separate and independent processes. Despite its limitations, such research is a useful source of information for evolutionary accounts.

Moreover, identifying when interactions between groups (e.g. bands) have become regular and important is not easy. It is a complex challenge to determine a social organization from archeological findings. There are reasons to suppose that inter-band cooperation has early origins, already in *heidelbergensis*, even if its evolution remained incomplete until late in human evolution and if in some contexts this interaction is not typical [21]. A complex social world almost certainly predates anatomically modern humans' last out of Africa migration [21]. As we learn more about the variability and flexibility in the social organization during human evolution, we may need to consider that the interactions of norm psychology and social identity were richer and important earlier than previously thought.

Another area that may benefit from the integration urged in this paper is the study of social complexity. Some theories define complex societies as those where groups are comprised social roles [64,65], and others view social complexity in terms of variations between and within social relationships [66]. Our discussion suggests that a key factor for understanding normative behaviour in complex societies with multiple roles, identities, allegiances and subgroups (e.g. ethnicities, political affiliations and religions), which shift and change over time, is the negotiation of social identities. Such negotiation occurs both between individuals and within a single individual, harbouring multiple, possibly conflicting, social identities and commitments. Observed social behaviour and surveys of attitudes tell a lot about norms and attitudes toward norms in society [67]. However, normative behaviour and attitudes towards norm following may also result from the complex interplay of norms with dynamic social identities and social contexts (including their expectations about the norm abidance of people they interact with). This can lead to a paradoxical mismatch between a society's degree of tightness and the degree of individuals’ commitment to the norms of the groups they belong to or identify with, and may explain why a country with many conservative groups appears to be relatively loose or vice versa (cf. [6,32]).

The framework outlined in this paper has implications for understanding major transitions in human evolution. Evolutionary processes have changed the structure and dynamics of group behaviour in human groups by shifting the focus from genetic relatedness to cultural elements and social institutions, which allowed complex societies to flourish. Complex societies comprise many different groups with different norms, whose members regularly interact with people from their out-groups. Current knowledge suggests that multi-level societies probably have early origins and have existed for a large part of human evolution. If so, social identity and social norms most likely affected each other. Understanding the interwoven connection between social norms and social identity, as well as people's commitment to both and their ability to navigate between them, are necessary for understanding how the transition from small-scale societies to complex, non-kin-related societies was possible in the first place.

## Data Availability

This article has no additional data.

## Endnote

1 It should be noted that social identity theory (SIT) and social categorization theory (SCT) are two distinct theories, although both capture the socially embedded group-located properties of human beings. Given that SCT was developed by John Turner, and he was also the co-developer of SIT (together with Henri Tajfel), there are substantial similarities between the two approaches, and in order to appreciate what is distinctive about SCT, it is necessary to some degree to examine aspects of SIT. A detailed discussion on the origins of SCT can be found in [14,25–27]. Having said that, the distinction between SIT and SCT is less relevant for the purpose of the current paper.

## References

[1] Migliano AB et al. 2017 Characterization of hunter-gatherer networks and implications for cumulative culture. Nat. Hum. Behav. **1**, 0043. (10.1038/s41562-016-0043)

[2] Szathmáry E. 2015 Toward major evolutionary transitions theory 2.0. Proc. Natl Acad. Sci. USA **112**, 10 104-10 111. (10.1073/pnas.1421398112) 25838283 PMC4547294

[3] Chung A, Rimal R. 2016 Social norms: a review. Rev. Commun. Res. **4**, 1-28. (10.12840/issn.2255-4165.2016.04.01.008)

[4] Chudek M, Henrich J. 2011 Culture-gene coevolution, norm-psychology and the emergence of human prosociality. Trends Cogn. Sci. **15**, 218-226. (10.1016/j.tics.2011.03.003) 21482176

[5] Bicchieri C. 2016 Norms in the wild: how to diagnose, measure, and change social norms. Oxford, UK: Oxford University Press.

[6] Gelfand M. 2018 Rule makers, rule breakers: how tight and loose cultures wire our world. New York, NY: Scribner.

[7] Henrich J. 2015 The secret of our success: how culture is driving human evolution, domesticating our species, and making us smarter. Princeton, NJ: Princeton University Press.

[8] Sripada CS, Stich S. 2006 A framework for the psychology of norms. In The innate mind, volume 2: culture and cognition (eds P Carruthers, S Laurence, Stephen Stich), pp. 280-320. Oxford, UK: Oxford University Press.

[9] Tomasello M. 2014 A natural history of human thinking. Cambridge, MA: Harvard University Press.

[10] Wrong DH. 1961 The oversocialized conception of man in modern sociology. Am. Sociol. Rev. **26**, 183-193. (10.2307/2089854)

[11] Bicchieri C, Muldoon R, Sontuoso A. 2018 Social norms. In The Stanford encyclopedia of philosophy [online] (ed. EN Zalta).

[12] Cancian FM. 1975 What are norms? A study of beliefs and action in a Maya community. Cambridge, UK: Cambridge University Press.

[13] Tajfel H. 1981 Human groups and social categories: studies in social psychology. Cambridge, UK: Cambridge University Press.

[14] Turner JC, Hogg MA, Oakes PJ, Reicher SD, Wetherell MS. 1987 Rediscovering the social group: a self-categorization theory. Oxford, UK: Blackwell.

[15] Henrich J, Gil-White FJ. 2001 The evolution of prestige: freely conferred deference as a mechanism for enhancing the benefits of cultural transmission. Evol. Hum. Behav. **22**, 165-196. (10.1016/S1090-5138(00)00071-4) 11384884

[16] Jiménez ÁV, Mesoudi A. 2019 Prestige-biased social learning: current evidence and outstanding questions. Palgrave Commun. **5**, 20. (10.1057/s41599-019-0228-7)

[17] McClanahan KJ, Maner JK, Cheng JT. 2021 Two ways to stay at the top: prestige and dominance are both viable strategies for gaining and maintaining social rank over time. Pers. Soc. Psychol. Bull. **48**, 1516-1528. (10.1177/01461672211042319) 34554036

[18] Chen Zeng T, Cheng JT, Henrich J. 2022 Dominance in humans. Phil. Trans. R. Soc. B **377**, 20200451. (10.1098/rstb.2020.0451) 35000450 PMC8743883

[19] Boyd R, Richerson P. 1992 Punishment allows the evolution of cooperation (or anything else) in sizable groups. Ethol. Sociobiol. **13**, 171-195. (10.1016/0162-3095(92)90032-Y)

[20] Henrich J. 2006 Cooperation, punishment, and the evolution of human institutions. Science **312**, 60-61. (10.1126/science.1126398) 16601179

[21] Sterelny K. 2021 The Pleistocene social contract. Oxford, UK: Oxford University Press.

[22] Andersson C, Czaran T. 2022 The transition from animal to human culture: simulating the social protocell hypothesis. Phil. Trans. R. Soc. B **378**, 20210416. (10.1098/rstb.2021.0416) PMC9869448 36688383

[23] Dor D. 2022 Communication for collaborative computation: two major transitions in human evolution. Phil. Trans. R. Soc. B **378**, 20210404. (10.1098/rstb.2021.0404) PMC9869436 36688385

[24] Parsons T. 1951 The social system. New York: NY: Routledge.

[25] Turner J et al. 1996 Henri Tajfel: an introduction. In Social groups and identity: developing the legacy of Henri Tajfel (ed. WP Robinson), pp. 1-24. Oxford, UK: Butterworth Heinemann.

[26] Turner J, Oakes PJ. 1997 The socially structured mind. In The message of social psychology (eds C McGarty, SA Haslam), pp. 355-373. Oxford, UK: Blackwell.

[27] Turner J, Reynolds K. 2010 The story of social identity. In Rediscovering social identity: core sources (eds T Postmes, N Branscombe), pp. 13-32. London, UK: Routledge.

[28] Turner J, Reynolds K. 2012 Self-categorization theory. In Handbook of theories of social psychology (eds PAM Van Lange, AW Kruglanski, E Tory Higgins), pp. 399-417. London, UK: SAGE Publications.

[29] Tajfel H, Turner JC, Austin WG, Worchel S. 1979 An integrative theory of intergroup conflict. Org. Identity **56**, 9780203505984-16.

[30] Bowles S, Gintis H. 2011 A cooperative species: human reciprocity and its evolution. Princeton, NJ: Princeton University Press.

[31] Tomasello M. 2009 Why we cooperate. Cambridge, MA: MIT Press.

[32] Gelfand M et al. 2011 Differences between tight and loose cultures: a 33-nation study. Science **332**, 1100-1104. (10.1126/science.1197754) 21617077

[33] Tajfel H. 1972 Social categorization. In Introduction a La Psychologie Sociale, Vol. 1 (ed. S. Moscovici). Paris, France: Larouse.

[34] Bornstein G, Ben-Yossef M. 1994 Cooperation in intergroup and single-group social dilemmas. J. Exp. Soc. Psychol. **30**, 52-67. (10.1006/jesp.1994.1003)

[35] Kramer RM, Brewer MB. 1984 Effects of group identity on resource use in a simulated commons dilemma. J. Pers. Soc. Psychol. **46**, 1044-1057. (10.1037/0022-3514.46.5.1044) 6737205

[36] Bicchieri C. 2005 The grammar of society: the nature and dynamics of social norms. Cambridge, UK: Cambridge University Press.

[37] Gintis H. 2016 Individuality and entanglement: the moral and material bases of social life. Princeton, NJ: Princeton University Press.

[38] Durkheim E. 1968 The elementary forms of the religious life. New York, NY: Dover Publications.

[39] Gavrilets S. 2020 The dynamics of injunctive social norms. Evol. Hum. Sci. **2**, e60. (10.1017/ehs.2020.58) PMC10427483 37588350

[40] Gavrilets S, Richerson PJ. 2017 Collective action and the evolution of social norm internalization. Proc. Natl Acad. Sci. USA **114**, 6068-6073. (10.1073/pnas.1703857114) 28533363 PMC5468620

[41] Gintis H. 2003 The hitchhiker's guide to altruism: gene-culture coevolution, and the internalization of norms. J. Theor. Biol. **220**, 407-418. (10.1006/jtbi.2003.3104) 12623279

[42] Henrich J, Ensminger J. 2014 Experimenting with social norms: fairness and punishment in cross-cultural perspective. New York, NY: Russell Sage Foundation.

[43] Achen C, Bartels L. 2016 Democracy for realists: why elections do not produce responsive government. Princeton, NJ: Princeton University Press.

[44] Akerlof G, Kranton R. 2000 Economics and identity. Q. J. Econ. **115**, 715-753. (10.1162/003355300554881)

[45] Chang D, Chen R, Krupka E. 2019 Rhetoric matters: a social norms explanation for the anomaly of framing. Games Econ. Behav. **116**, 158-178. (10.1016/j.geb.2019.04.011)

[46] Chen Y, Li SX. 2009 Group identity and social preference. Am. Econ. Rev. **99**, 431-457. (10.1257/aer.99.1.431)

[47] Hogg M, Abrams D, Brewer MB. 2017 Social identity: the role of self in group processes and intergroup relations. Group Process. Intergroup Relations **20**, 570-581. (10.1177/1368430217690909)

[48] Kelly D, Davis T. In press. A framework for the emotional psychology of group membership. Rev. Phil. Psych. (10.1007/s13164-021-00561-6)

[49] Mason L. 2018 Uncivil agreement: how politics became our identity. Chicago, IL: University of Chicago Press.

[50] Smaldino P. 2019 Social identity and cooperation in cultural evolution. Behav. Processes **161**, 108-116. (10.1016/j.beproc.2017.11.015) 29223462

[51] Pickup MA, Kimbrough E, de Rooij EA. 2020 Identity and the self-reinforcing effects of norm compliance. Southern Econ. J. **86**, 1222-1240. (10.1002/soej.12410)

[52] Westra E, Andrews K. 2021 A new framework for the psychology of norms. *Ethics and Psycholog*y, 29 September. (10.31234/osf.io/aqv8c)

[53] Jordan F et al. 2013 Cultural evolution of the structure of human groups. In Cultural evolution: society, technology, language, and religion (eds PJ Richerson, MH Christiansen), pp. 87-116. Cambridge, MA: MIT Press.

[54] Richerson P et al. 2016 Cultural group selection plays an essential role in explaining human cooperation: a sketch of the evidence. Behav. Brain Sci. **39**, e30. (10.1017/S0140525X1400106X) 25347943

[55] Richerson P, Henrich J. 2009 Tribal social instincts and the cultural evolution of institutions to solve collective action problems. *Context and the Evolution of Mechanisms for Solving Collective Action Problems Paper*, available at: https://ssrn.com/abstract=1368756 or http://dx.doi.org/10.2139/ssrn.1368756.

[56] Richerson PJ, Boyd R. 2001 The evolution of subjective commitment to groups: a tribal instincts hypothesis. Evol. Capacity Commitment **3**, 186-220.

[57] Townsend C. 2018 Egalitarianism, evolution of. In The Wiley Blackwell international encyclopedia of anthropology. New York, NY: John Wiley and Sons.

[58] Bird DW, Bird RB, Codding BF, Zeanah DW. 2019 Variability in the organization and size of hunter-gatherer groups: foragers do not live in small-scale societies. J. Hum. Evol. **131**, 96-108. (10.1016/j.jhevol.2019.03.005) 31182209

[59] Tomasello M. 2019 The moral psychology of obligation. Behav. Brain Sci. **43**, e56. (10.1017/S0140525X19001742) 31133086

[60] Singh M, Glowacki L. 2022 Human social organization during the Late Pleistocene: beyond the nomadic-egalitarian model. Evol. Human Behav. **43**, 418-431. (10.32942/osf.io/vusye)

[61] Evans N. 2017 Did language evolve in multilingual settings? Biol. Philos. **32**, 905-933. (10.1007/s10539-018-9609-3)

[62] Scott JF. 1971 Internalization of norms: a sociological theory of moral commitment. Hoboken, NJ: Prentice-Hall.

[63] Zefferman M. 2014 Direct reciprocity under uncertainty does not explain one-shot cooperation, but demonstrates the benefits of a norm psychology. Evol. Hum. Behav. **35**, 358-367. (10.1016/j.evolhumbehav.2014.04.003)

[64] Kappeler PM, Clutton-Brock T, Shultz S, Dieter L. 2019 Social complexity: patterns, processes, and evolution. Behav. Ecol. Sociobiol. **73**, 1-6. (10.1007/s00265-018-2618-z)

[65] Rubenstein DR, Abbot P. 2017 Social synthesis: opportunities for comparative social evolution. In Comparative social evolution (eds DR Rubenstein, P Abbot), pp. 427-452. Cambridge, UK: Cambridge University Press.

[66] Aureli F, Schino G. 2019 Social complexity from within: how individuals experience the structure and organization of their groups. Behav. Ecol. Sociobiol. **73**, 1-13. (10.1007/s00265-018-2604-5)

[67] Gelfand MJ, Harrington JR, Jackson JC. 2017 The strength of social norms across human groups. Perspect. Psychol. Sci. **12**, 800-809. (10.1177/1745691617708631) 28972845

